# Dental caries prevention strategies among children and adolescents with immigrant - or low socioeconomic backgrounds- do they work? A systematic review

**DOI:** 10.1186/s12903-018-0478-6

**Published:** 2018-02-07

**Authors:** Marit S. Skeie, Kristin S. Klock

**Affiliations:** 1 Department of Clinical Dentistry, Pediatric Dentistry, The Faculty of Medicine, University of Bergen, Aarstadveien 19, N-5009 Bergen, Norway; 2 Department of Clinical Dentistry, Community Dentistry, The Faculty of Medicine, University of Bergen, Aarstadveien 19, N-5009 Bergen, Norway

**Keywords:** Dental caries, Socioeconomy, Children, Immigrants, Prevention, Systematic literature review

## Abstract

**Background:**

This systematic review was designed to uncover the most reliable evidence about the effects of caries preventive strategies in children and adolescents of immigrant or low socioeconomic backgrounds.

**Methods:**

According to pre-determined inclusion and exclusion criteria, relevant articles focusing on underprivileged groups were electronically selected between January1995 and October 2015. The literature search was conducted in five databases; PubMed, Embase, CINAHL, SweMed+ and Cochrane Library. Accepted languages for included articles were English, German and Scandinavian languages. Abstracts and selected articles in full text were read and assessed independently by two review authors. Systematic reviews and meta-analyses were not included. Also articles with topics of water fluoridation and fluoride toothpaste were excluded, this due to all existing evidence of anti-caries effect for disadvantaged groups. The key data about the main characteristics of the study were compiled in tables and a quality grading was performed.

**Results:**

Thirty-seven articles were selected for further evaluation. Supervised toothbrushing for 5-year-old school children was found to be an effective prevention technique for use in underprivileged groups. Also a child/mother approach, targeting nutrition and broad oral health education of mothers showed effectiveness. For older children, a slow-release fluoride device and application of acidulated phosphate fluoride (APF) gel showed to be effective.

**Conclusion:**

On the basis of this review, we maintain that in addition to studies of water fluoridation and fluoride toothpaste, there are other preventive intervention studies providing scientific evidence for caries reduction among children and adolescents with immigrant or low socioeconomic backgrounds.

## Background

Dental caries is a disease with modifiable risk factors that during recent decades has been shown to have a skewed distribution in both the industrialized and the non-industrialized world. Those most heavily affected are usually found in vulnerable population groups as among people with low income, in some immigrant minorities and where these two populations overlap [1]. This information is today so widely accepted that Early Childhood Caries (ECC) is used as a marker of social inequality [2]. Furthermore, the general caries decline, documented among children and adolescents, has mostly benefitted the general population and has been shown to be limited in peers from low-income households or from ethnic minority groups [3–7].

Epidemiological studies from the Scandinavian countries (Denmark, Sweden and Norway) have revealed disparities in caries between children and adolescents with and without foreign backgrounds [8–10] and concluded a worse caries status among those with immigrant background, this in accordance with recent findings from USA [11]. Among caries determinants, not only low income, but also socio- cultural conditions that influence their lives, play important roles [8]. To strength their identity, it is not unusual that unfavorable types of diet and dietary patterns from country of origin are maintained in its traditional way [12].

Different theoretical explanations for inequality in oral health have been discussed and presented in a literature review by Sisson in 2007 [13]. According this author, future research should focus more on how people live their lives in different social classes and how background social factors influence lifestyle decisions [13]. Lack of understanding of individuals in their social context, how they live their lives, may be one of the multi-faceted reasons why traditional caries prevention programs have not succeeded in disadvantaged groups. It is understandable that prevention of oral diseases is not given the highest priority under challenging living circumstances. Young people living in vulnerable population groups tend to have parents with low educational backgrounds, which entails a higher risk of having negative attitudes concerning their children’s oral health related behaviours [14]. Having diets with high sugar content and sub-optimal tooth brushing habits are associated with some immigrant subgroups [15, 16] and with materially deprived neighbourhoods [17]. For the poorest families, the cost of toothbrushes and toothpaste may represent a potential barrier to regular toothbrushing [18]. In some countries, lack of available and accessible regular dental care constitutes a barrier and, especially in those countries, the children who need dental care the most are the ones least likely to visit a dental clinic [19].

Difficulty reducing disparities in oral health is a matter of concern for researchers worldwide [20]. Traditional interventions are criticized for lacking or not sufficiently considering the socio-economic context [21] or, additionally, the oral health impact of acculturation [22]. Adjustment of established preventive strategies or formation of new ones are needed [23, 24] which, in focusing on living conditions and lifestyles, should combine social policy and individual actions [20]. In spite of this, traditional interventions with wide-ranging approaches, still play their roles in reducing oral health inequalities, as all individuals share many risk factors [25].

Caries is a largely preventable disease. In order to reach the goal of reducing oral health inequalities [19, 26], it is essential to search for scientific evidence for effectiveness in preventive programs targeting disadvantaged and socially marginalized groups. These groups have a history of not benefitting from traditional preventive interventions [27]. However, to our knowledge, there are few literature reviews concerning a caries preventive intervention approach to children and adolescents from subgroups of immigrant populations or from low socioeconomic backgrounds. For this reason, the aim of this systematical review is to uncover the most reliable evidence about the effects of caries preventive strategies in children and adolescents of immigrant or low socioeconomic backgrounds. The research question we wish to answer is: “Do preventive strategy studies exist that offer scientific evidence for caries reduction among children and adolescents with immigrant or low socioeconomic backgrounds?”

## Methods

On March 2nd 2015, a systematic electronic literature search was conducted in five databases; PubMed, Embase, CINAHL, SweMed+ and Cochrane Library. Articles were also identified through hand searches in the reference lists of already selected articles. Totally, 1804 abstracts were identified. An updated search was undertaken 10 Oct the same year, but no other articles were found or included in the final list. Table 1 shows the search equation applied in terms of MeSH terms and search words for each database.

**Table:** **Table 1**

PubMed	
The free text in PubMed translated and combined with MeSH-terms (Medical Subjects Headings (MeSH): (patient education OR health education dental OR prevention OR promotion OR motivation OR motivating interview OR Program evaluation OR Dental care for children) AND (immigrant OR immigrants OR refugee OR refugees OR socioeconomic factors OR vulnerable OR Indigent OR indigency OR poverty) AND dental caries AND (child OR children)	
EMBASE	
((patient education or health education dental or prevention or promotion or motivation or motivating interview or Program evaluation or Dental care for children) and (immigrant or immigrants or refugee or refugees or socioeconomic factors or vulnerable or Indigent or indigency or poverty) and dental caries and (child or children)).mp. Explanation for searching fields: [mp = title, abstract, heading word, drug trade name, original title, device manufacturer, drug manufacturer, device trade name, keyword]	
Cinahl	
((patient education or health education dental or prevention or promotion or motivation or motivating interview or Program evaluation or Dental care for children) and (immigrant or immigrants or refugee or refugees or socioeconomic factors or vulnerable or Indigent or indigency or poverty) and dental caries and (child or children))	
SweMed+	
Search with MeSH-terms.	
Search no.	Search words
1	exp: “dental caries”
3	exp: “socioeconomic factors”
8	exp: “emmigrants and immigrants”
10	#3 OR #8
13	exp: “Infant” OR “child” OR “adolescent”
14	#1 AND #10 AND #13
Cochrane	
ID	Search
#1	MeSH descriptor: [Dental Caries] explode all trees
#2	Caries
#3	MeSH descriptor: [Socioeconomic Factors] explode all trees
#4	migrant* or immigrant* or refugee*
#5	child or children
#6	#1 or #2
#7	#3 or #4
#8	#5 and #6 and #7

The search was limited to peer-reviewed articles and it did not allow systematic reviews nor meta-analyses to be included. Only articles published during the period January1995 - October 2015 were included. Accepted languages for included articles were English, German, Norwegian, Swedish or Danish. Non-randomized studies were accepted. Grey literature was excluded as this type of literature can vary considerably in standard of quality, review and production. Otherwise, the search algorithm included many ways of grouping socio-demographic-economic backgrounds, therefore different models for this were revealed in the articles; education or profession of the parents or caregivers, socially deprived or low income communities, child’s home post code, assigned as a Carstairs socio-economic deprivation score and others scores such as the Jarman underprivileged Area score, Townsend index and the ABIPEME index. The term “Underprivileged group” was used to cover all aspects used in the articles, but articles based on populations from explicit indigenous or tribe groups were excluded. The studies described in the articles were divided into national, subnational (from regions) and community (cities or small areas) levels.

Studies with a clear outcome measure like caries experience or caries prevalence were included, but not studies comparing different caries interventions without a control group. Interventions were denoted as effective when statistically significant differences in dental caries status could be documented between intervention- and control groups. In cases where other outcome measures together with caries experience were investigated, these measures were not included as outcomes in the tables and their results were also not reported.

**Fig. 1:**
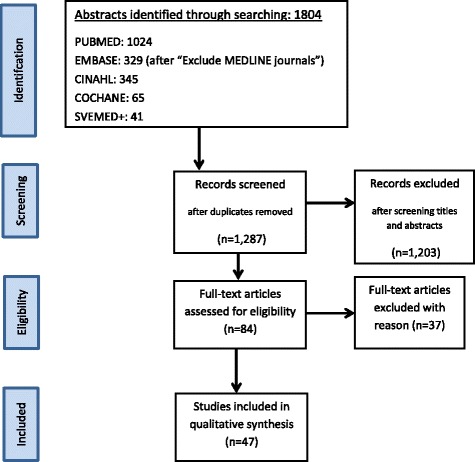
Flow diagram showing the literature search strategy (flow chart adapted from Mejare et al. 2015 [55]

The different steps of a PRISMA [28] (Fig. 1) were used as a platform when performing the systematic literature review (identification, screening, eligibility, inclusion). The identification consisted of three levels in which inclusion and exclusion criteria were followed: 1) title and authors, 2) abstract and 3) full text. During the whole searching process until the final results, the same two review authors (MSS, KSK) independently read and evaluated the articles. Printed articles were only read when abstracts were determined to be of relevance and within the scope of the present systematic review. Later, both review authors independently did the assessment before deciding to include the article into the final review. If, for example, only one author found the abstract potentially relevant, but the other did not, full-text articles were read by both. In case of further disagreement, extra time to discuss was used until a consensus was reached. As both authors agreed on the selection, a consensus was not necessary. The articles not included did not undergo further analysis. Altogether, 37 articles were selected. As both water fluoridation [29, 30] and fluoride toothpaste [31, 32] for years have presented evidence that they provide anti-caries benefit for disadvantaged groups, it was seen upon as superfluous and unnecessary for further evaluation in this literature review.

The PICOS approach was followed to give the reader the key information (participants, interventions, comparators, and study design). These central themes and topics were extracted from the included articles and presented in tables. The Hierarchy of research design of the U.S. Preventive Services Task Forces (USPSTF) [33] was used to grade the level of quality of evidence (Table 2), but additional information was added about sample size, randomization, caries examination calibration, number of examiners, radiographic examination, blinding and outcome measures. Evidence based on randomized controlled trials begins as high quality evidence, but the confidence in evidence gradually diminished for several reasons, including:1) study limitations; 2) inconsistency of results; 3) indirectness of evidence; 4) imprecision; and 5) reporting bias [34]. The final discussion, summarizing the evidence for effectiveness, included only randomized study designs. To define age distribution of various studies, the studies were categorized to either belong to Age group I: mother/baby approach/pre-school children at baseline (≤5-yr-olds), or Age group II: schoolchildren and adolescents.

**Table 2:** Quality of evidence according to assessment system of the US Preventive Services Task Force, from Grimes and Schulz [33]

I	Evidence from at least one properly designed randomized control trial
II-1	Evidence obtained from well-designed controlled trials without randomization
II-2	Evidence from well-designed cohort or case-control studies, preferably from more than one centre or research group
II-3	Evidence from multiple time series with and without the intervention. Important results in uncontrolled experiments (such as introduction of penicillin treatment in the 1940s) could also be considered as this type of evidence
III	Opinions of respected authorities, based on clinical experience, descriptive studies, or reports of expert committees

## Results

Thirty-seven articles were selected for further evaluation. The key data about the main characteristic of the studies are compiled in Table 3. A wide range of intervention domains were presented; “Other fluoride supplements” (*n* = 9), “Oral health studies and programs including fluoride supplements with other intervention types” (*n* = 13), “Sealants” (*n* = 3), “Supervised toothbrushing” (*n* = 3), “Nutrition” (n = 3), “Motivating interviewing” (*n* = 1), “Oral health education” (*n* = 4) and “Remineralizing paste” (*n* = 1). Different countries were represented in the extracted publications, but the majority (*n* = 24) originated from European countries, especially from Great Britain. Fewer articles were published before 2009 than more recently, in 2009 and after (16 *vs*19 articles).

**Table 3:** Studies’ characteristics (*n* = 37). Studies focusing on water fluoridation and fluoride toothpaste are not included

# Study	Country Level	Age groups (yrs)	Follow – up/retrospective period	Intervention
Other fluoride supplement (n = 9)				
1. Agrawal N & Pushpanjali K. 2011 [40]	India Community	9–16	Follow-up: 6/12 mo.	Acidulated phosphate fluoride (APF) gel
2. Meyer-Lueckel H et al. 2010 [56]	Germany Community	6–9	Retrospective period: 2–4 yrs.	Fluoride tablets
3. Oliveira BH et al. 2014 [57]	Brazil Community	1–4	Follow up: 2 yrs.	Fluoride Varnish
4. Pitchika V et al. 2013 [58]	Germany Subnational	2–3	Follow-up: 2 yrs.	Fluoride Varnish
5. Schuller AA & Kalsbeek H. 2003 [59]	The Netherlands Subnational	15–17	Cross-sectional comparisons	Topical fluoride
6. Riley JC et al. 2005 [60]	United Kingdom Subnational	Mean age: 10–11	Cross-sectional comparisons	Milk fluoridation
7. Levin KA et al. 2009 [61]	United Kingdom Subnational	Mean age: 11.39	Cross-sectional comparisons	Fluoride rinsing
8. Toumba KJ & Curzon ME. 2005 [41]	United Kingdom Community	8	Follow up: 2 yrs	Slow-releasing fluoride device
9. Wennhall I et al. 2014 [62]	Sweden Community	12–14	Follow up: 2 yrs	Fluoridated salt
Oral health studies and programs including fluoride supplements with other intervention types (*n* = 13)				
10. Minah G et al. 2008 [63]	USA Community	6–27 mo	Follow up: 26 mo	For high caries risk subjects with caries experience and high MS units: fluoride varnish and reinforcement of caries prevention
11. Bravo et al. 1997 [42]	Spain Community	6–8	Follow up: I yr	Sealant and Fluoride Varnish
12. Songpaisan Y et al. 1995 [64]	Thailand Community	7–8, 12–13	Follow up: 2 yrs	GI cement/sealants/HF application
13. Wennhall I et al. 2008 [65]	Sweden Subnational	2	Follow up: 3 yrs	Parent education/ toothbrushing instruction/ diet/fluoride tablets
14. Wagner Y et al. 2014 [66]	Austria Subnational	New mothers at time after birth	Retrospective evaluation of outcome of Oral health Promoting Program - children 5-yrs	Oral hygiene instructions for mother/child (MI approach)
15. Dülgergil CT et al. 2005 [67]	Turkey Community	10–11	Follow up: 6 mo, 1 yr	ART in combination with Fluoride Varnish fissure sealants
16. Meurman P et al. 2009 [68]	Finland Community	18 mo	Follow up: 5-yrs.	For families for MS-positive children: Health education to caretakers/ xylitol lozenges for the child.
17. Armfield JM & Spencer AJ. 2007 [43]	Australia Subnational	Mean age: 10.5	Follow-up: mean of 2 yrs	Fissure sealants in combination with water fluoridation
18. Blair Y et al. 2004 [69]	Scotland Community	36–59 mo	Follow-up: 2 yrs., 4 yrs	Community based oral health program; nutrition, oral hygiene, fluoride dentifrice, outreach activity
19. Blair Y et al. 2006 [70]	Scotland Community	5	Follow-up: 6 yrs. Secondary analysis of routine caries datasets 1997–98 to 2003–04	Community based oral health program
20. Stokes E et al. 2011 [44]	Great Britain Community	13	Follow-up: 2 yrs.	Supervised toothbrushing/self-applied high-fluoride gel by toothbrushing
21. Lindgard M 2013 [71]	Sweden Community	2	Follow-up: 1 yr.	Prevention program-oral information, diet and hygiene counseling, fluoride tablets
22. Baca P et al. 2004 [72]	Spain Community	6–7	Follow-up: 24 mo	Clorhexidine in combination with Thymol Varnish
Sealants (*n* = 3)				
23. Muller-Bolla M et al. 2013 [73]	France Community	6–7	Follow-up: 1 yr.	Resin-based sealant
24. Tickle M et al. 2007 [74]	England Subnational	5–14	Retrospective period: data collected from patients’ case notes	Fissure sealant
25. Baldini V et al. 2010 [75]	Portugal Sub- national	8	Retrospective period: 2 yrs. (assessed when 10 yrs)	Sealant
Toothbrushing (*n* = 3)				
26. Curnow MM et al. 2002 [38]	Scotland Subnational	Mean age 5.3.	Follow-up: 2 yrs	Supervised toothbrushing
27. Jackson RJ et al. 2005 [39]	England Community	Mean age: 5.63	Follow-up: at 21 mo	Supervised toothbrushing
28. Macpherson LMD et al. 2013 [52]	Scotland National	5	Population study involving multiple cross-sectional dental epidemiology surveys	Supervised toothbrushing
Nutrition (*n* = 3)				
29. Freeman R et al. 2001 [76]	Ireland Subnational	9	Follow-up: 1 yr., 2 yrs	Healthier eating (BBB)
30. Feldens CA et al. 2010 [36]	Brazil Community	6, 8, 10, 12 mo	Follow-up: 4 yrs.	Nutritional program (mother/child approach)
31. Chaffee BW et al. 2013 [77]	Brazil Community	6 mo	Follow-up:12 mo, 36 mo	Nutritional training (mother/child approach)
Motivating Interviewing (*n* = 1)				
32. Ismail AL et al. 2011 [78]	USA Community	0–5	Follow-up: 2 yrs.	Tailored motivational intervention (mother/child approach)
Oral health education (*n* = 4)				
33. Kressin NR et al. 2009 [79]	USA Community	6mo- 5 yrs	Follow-up	For parents: Communications skills training/ EMR/educational brochure
34. Kowash MB et al. 2000 [80]	United Kingdom Community	11.4 mo	Follow-up: 3 yrs	Oral health long term education programme
35. Mohebbi SZ et al. 2009 [37]	Iran National	12–15 mo with mothers	Follow-up: 6 mo	Educational intervention
36. van Palenstein Helderman WH et al. 1997 [81]	Tanzania Community	9–14 yrs	Follow-up: 3,8,15 and 36 mo	School-based OHE programme; education and supervised toohbrushing
Remineralizing Paste (*n* = 1)				
37. Plonka KA et al. 2013 [82]	Australia Subnational	6 mo	Follow-up: 12, 18, 24 mo	Comparing a remineralizing paste with and antibacterial gel

Table 4 is a quality rating of the different studies including topics such as sample size, randomization, caries examination calibration, number of oral examiners, use of radiographic technologies, blinding, length of intervention period and type of outcome measurement. The overall judgement of the quality of evidence showed that few studies (*n* = 3) used radiographic bitewings. Only six studies explicitly reported that they included enamel caries in the caries examination. Table 5, based on the USPSTF evidence classification and modified by Tutak M et al. [35], illustrates that the intervention target age groups differed; 19 studies belonged to Age group I and 18 studies to Age group II. Articles categorized separately as Level of evidence I (from properly designed randomized control trial) were confined both to Age group I (*n* = 8) and Age group II (*n* = 6). Four studies of the originally Level of evidence I studies provided evidence of caries reduction in Age group I. The domains were “Nutritional program” in a mother and child approach (*n* = 1) [36], “Oral health educational intervention program” (n = 1) [37] and “Supervised toothbrushing” (*n* = 2) [38, 39]. The sample sizes of the two last mentioned studies were pooled, resulting in a sample size of 831 (completing the trial: test = 420, controls = 411).

**Table 4:** Description of various ways of characterizing the studies. The Level of evidence by US preventive services task forces hierarchy of research design is used

# Study	Level of evidence	Sample Size ^a^	Randomization	Caries examination calibration	No of oral examiners	Radiographic examination	Blinding	Outcome measurement	Results ^b^Effectiveness
1. Agrawal N & Pushpanjali K [40]	I	257	Yes	Yes	2	No	Yes	DMFT/S and Incipient lesions (IL)	Effective for IL only
2. Meyer-Lueckel H et al. [56]	II-2	583	No	Yes	1	No	Yes	Modified ≥defs	Effective (children: low caries levels)
3. Oliveira BH et al. [57]	I	200	Yes	Yes	2	No	Yes	Pitts et al.: ICDAS (d_2_mfs d_3_mfs)	Not effective
4. Pitchika V et al. [58]	II-1	215^a^	No	Yes	1	No	No	WHO: d_1–2_ s d_3-4_mfs	Effective for d_1–2_ s only
5. Schuller AA & Kalsbeek H 2003 [59]	II-2	745	No	Not reported	3	Yes	Yes	D _1–3_ s FS	Not effective
6. Riley JC et al. [60]	II-2	2525	No	Yes	2	No	Not reported	DMFT, DT, DFS	Effective
7. Levin KA et al. [61]	II-2	1333	Yes	Yes	3	No	Yes	D_3_MFT	Effective
8. Toumba KJ & Curzon ME. [41]	I	174	Yes	Yes	1	No	Yes	Palmer et al.: (dmft/s, DMFT/S)	Effective
9. Wennhall I et al. [62]	II-1	733	No	Yes	2	Yes	Not reported	WHO: DFS increment (enamel caries included)	Not effective
10. Minah G et al. [63]	II-2	219	No	yes	2	No	Not reported	dmfs, precavitated lesions, MS	Effective
11. Bravo et al. [42]	I	314	Yes	Not reported	1	No	Yes	WHO: DMFS	Effective
12. Songpaisan Y et al. [64]	II-2	1110	No	Not reported	1	No	Not reported	DFS increment	Effective in some groups
13. Wennhall I et al. [65]	II-1	852	No	Yes	2	Yes	Not reported	defs/deft	Effective
14. Wagner Y et al. [66]	II-2	471	No	yes	2	No	Not reported	WHO: d_3_mfs/ d_3_mft/ care index	Effective
15. Dülgergil CT et al. et al. [67]	II-2	27, 147 lesions	No	Yes	2	No	Not reported	Modified Nyvad et al.: Mean dft/dfs/DMFT/DMFS	Effective
16. Meurman P et al. [68]	II-1	794	No	Yes	2	No	Not reported	dmft	Effective (children of white collar families, not blue-collars)
17. Armfield JM & Spencer AL [43]	I	789	Yes	No	Large number	No	Not reported	WHO: dmfs/DMFS	Effective
18. Blair Y et al. [69]	II-2	1553	No	Yes	Not reported	No	Not reported	BASCD: dmft	Effective
19. Blair Y et al. [70]	II-2	3506	Yes	Training	Not reported	No	Not reported	d_3_mft	Effective
20. Stokes E et al. [44]	I	473	Yes	Yes	1	No	Yes	Pitts: D_1_FS/D_3_FS	Effective for D_3_FS outcome
21. Lindgard M [71]	II-2	313	No	No	5	No	No	deft, enamel caries included	Effective
22. Baca P et al. [72]	II-2	181	Yes	yes	2	No	No	WHO: dftm/dfsm and DMFT	Effective
23. Muller-Bolla M et al. [73]	II-2	253 (421 pair of molars)	Yes	Yes	Not reported	No	No	ICDAS: increment. (code 3–6)	Effective
24. Tickle M et al. [74]	II-3	677	No	Yes	Not reported	Not reported	No	OR measurement: association between sealants and caries	Not effective
25. Baldini V et al. [75]	II-3	277	No	Yes	1	No	No	WHO: DMT increment	Effective
26. Curnow MM et al. [38]	I	461	Yes	Yes	1	No	Yes	ICDAS	Effective
27. Jackson HM et al. [39]	I	362	Yes	Yes	2	No	Yes	BASCD criteria. d_3_mfs Caries increment	Effective
28. Macpherson LMD & Conway DI. [52]	II-3	99,071 (7% and 25% (4472–12,716) National	No	Not reported	Not reported	No	Not reported	d_3_mft	Effective
29. Freeman R et al. [76]	II-1	238	No	Yes	1	No	Yes	BASCD criteria	Moderate effective (only for mean number of sound teeth)
30. Feldens et al. [36]	I	340	Yes	Yes	1	No	Yes	NIH (Drury). Occurence of ECC, S-ECC	Effective
31. Chaffee BW et al. [77]	I	458	Yes	Yes	2	No	Not reported	WHO: involving also non-cavitated lesions	Not effective (only a subgroup)
32. Ismail AI et al. [78]	I	599 caregiver/child	Yes	Yes	3	No	Not reported	ICDAS	Not effective
33. Kressin NR et al. 2009 [79]	II-2	1087	No	Not reported	Not reported	No	Yes	EC as cavitated lesions	Effective
34. Kowash MB et al. [80]	II-2	234 mother/child pairs	Yes	Yes	1	No	Not reported	(Palmer et al. involving also non-cavitated lesions	Effective
35. Mohebbi SZ et al. [37]	I	242 child/mothers	Yes	Yes	2	No	Yes	WHO: d_3_mft	Effective
36. van Palenstein et al. [81]	I	309 + 122	Randomly selected schools	Yes	1	No	Yes	Mean DMFT (WHO criteria	No effect
37. Plonka KA et al. [82]	I	542	Yes	Yes	2	No	Yes	Percentage of children with ECC	No effect

^a^: sample size restricted to SES information. ^b^: effectiveness, defined as significant caries reduction in the intervention group

**Table 5:** A categorization of the articles based on the US Task Force evidence classification, modified by Tutak M et al. [35]

US Task Force Level of evidence	Study design	No (%) of published articles	Bibliography numbers
Age group I. Mother/baby approach/pre-school children at baseline (≤5-yr- olds) (*n* = 19)			
I:	Randomized controlled trials	8 (42%)	[36–39, 57, 77, 78, 82]
II-1:	Controlled trials without randomization	3 (16%)	[58, 65, 68]
II-2:	Cohort or case-controlled studies	7 (37%)	[63, 66, 69–71, 79, 80]
II-3:	Case series	1 (5%)	[52]
III	Case reports, Opinions of authorities	0	
Age group II. Schoolchildren and adolescents (*n* = 18)			
I:	Randomized controlled trials	6 (33%)	[40–44, 81]
II-1:	Controlled trials without randomization	2 (11%)	[62, 76]
II-2:	Cohort or case-controlled studies	8 (45%)	[56, 59–61, 64, 67, 72, 73]
II-3	Case series	2 (11%)	[74, 75]
III	Case reports, opinions of authorities	0	

As for Age group II, five Level of evidence I studies reported having a caries reducing effect [40–44], though the intervention used by Agrawal et al. [40] showed effectiveness restricted to incipient lesions. The domains were “Other fluoride supplement” (*n* = 2) and “Oral health studies and programs including fluoride supplements with other intervention types” (*n* = 3).

## Discussion

The present systematic review targeted high caries risk groups with a history of not benefitting from traditional preventive interventions [27]. As reducing the burden of oral disease in poor and marginalized populations is within the framework of the WHO Oral Health Programme [20], the need for such a systematic review should be evident enough. Khan et al. have stressed that the adjective systematic imply a lot; clearly formulated questions, identification of relevant studies, quality assessment and summarizes of the evidence by use of explicit methodology [45]. Also the present posed research question, “Do studies of preventive strategies exist that present scientific evidence for caries reduction among children and adolescents with immigrant or low socioeconomic backgrounds?”, showed relevance, and should be described as both structured and explicit. According to Khan et al. the framing question fulfilled the requirements of Step 1 for conducting a systematic review. With respect to Khan et al., the present search based on five databases, though with some language restriction, to some extent fitted Step 2 of identifying relevant work.

This review was not the first to seek evidence of effectiveness for caries prevention. A recent review had an identical focus on ECC in the general child population [46], not restricted to underprivileged groups. The authors of another ECC review [47] concluded that their results reinforced the need for high quality clinical research conducted on different social and ethnic groups. Despite quality limitations in clinical research, reviews of evidence should continue to be undertaken, so that all selected studies should be subjected to as comprehensive, objective and attentive to quality assessment as possible.

To meet an evidence-based approach, the hierarchical grading system of USPSTF was used, but, due to its limitations referred in a paper of third USPSTF [33], this was not the only approach. In that paper it was claimed that a well-designed cohort study might be more compelling than an inadequately powered or poorly conducted randomized controlled trial. Randomized controlled trials have drawbacks too [48]. Due to this, in the present paper additional information was generated and presented in the different tables. Factors covered included the duration of the studies, sample sizes, calibration methods, number of examiners, blinding options, use of radiographic examination and outcome measurement according to caries diagnostic systems. This detailed quality overview should explore the heterogeneity of the included studies and thus permit a sufficiently critical evaluation.

The outcome, the effectiveness judged by caries reduction, must be analysed with caution due to its dependence on some underlying factors. One factor that showed heterogeneity was the outcome measurement. The dmtf/DMFT index was most often used, but whether enamel caries was included in the assessment varied. Another variation was the use of visual-tactile examination alone or the supplementary use of bitewing radiographs. Some studies also reported that caries diagnostic systems capable of diagnosing enamel lesions were used, but it was not always clearly explained whether enamel caries coding was used. Only three studies reported use of radiographs. This means that many studies not applying bitewing radiographs could have underscored proximal caries lesions [49]. Depending on the impact of these shortcomings of homogeneity, it was uncertain whether they had influenced the outcome effect or not, as the comparing groups (intervention and control groups) were both subjected to the same measurement methods. Furthermore, some studies had many examiners, which could have influenced the quality negatively. A previous systematic review of literature regarding methods for caries detection by Twetman et al. went as far as to exclude any trial with more than one examiner [50]. Some articles in the present review had a single or few examiners, but precise descriptions about how the caries calibration process had been conducted, important information [51], were often not described. However, also in these matters, what was most important was that the same examiners were responsible for caries registrations in both test and control groups.

The most evident finding from the present review was that those articles being classified as randomized controlled trials (Level I according to USPSTF) provided the best research evidence in other aspects like blinding, examiner calibration and number of examiners. Those articles that focused on oral health programs in which fluoride use constituted a component (domain: “Oral health studies and programs including fluoride supplements with other intervention types”), made it impossible to determine which intervention in the program was responsible for the effect. In Age group II, this problem affected three studies [42–44], leaving only two studies, those of Agrawal & Pushpanjali [40] and of Toumba & Curzon [41] for further evaluation (domain: “Other fluoride supplement”). The first study from India of Agrawal & Pushpanjali [40] was a community intervention trial, conducted to assess the feasibility of an acidulated phosphate fluoride (APT) gel as a caries-preventive agent in a high-risk group of school children (9–16-year-old) with a low socio-economic background. The follow-up period was short (1 year), but it reported a caries reduction for the APF gel. This short time effect might be considered a strength of the study and supports the intervention type. The study of Toumba and Curzon [41] had as its objective to test a fluoride-containing slow-released device in preventing dental caries in a group of low socio-economic schoolchildren from the age of 8 years during a 2-year-period. The authors concluded that the device attached to the buccal surface of the right maxillary first permanent molar, reduced caries incidence. As a consequence, the authors concluded that a fluoride-containing slow-released device showed promise as an preventive technique for use in schoolchildren and other priority groups.

Two studies in Age group I, targeting 5-year-olds, by Curnow et al. [38] and Jackson et al. [39]*,* showed valid findings. Both studies focused on the efficacy of supervised fluoride toothbrushing, targeted into socially deprived areas. A pooling of participants was possible due to the homogeneity of the studies (age, follow-up period, in advance training for the persons in charge of the toothbrushing, a single calibrated caries examiner, examiner blinding), and this made the sample size substantial. Both studies could show to a significant reduction in dental caries. A national supervised toothbrushing program has also verified that this sort of preventive program shows caries reduction [52]. Although the data from these articles were derived from children who commenced their preventive activities as 5-year-olds, and not during the first two years of life, as suggested to be the most important age for effective interventions [53], cost-effectiveness could be expected to be better than for older individuals in Age group II.

Two other studies, shown to be effective in reducing ECC in the targeted populations, were the study of Feldens et al. [36] and that of of Mohebbi et al. [37], as they involved interventions during the first years of life. The intervention domains were “Nutrition” and “Oral health education” respectively and had a mother/child approach with baseline when the child was very young (six months in the first study and 12 months in the other). Both used trained non-dental staff, respectively undergraduate nutrition students and trained health staff at health centres. As feeding habits and sugar intake were given high priority in the oral health education study as well, the two studies had many features in common. The study of Mohebbi et al., [37] conducted in Iran, had the shortest follow-up period, only 6- month-period, which reported in literature to be the shortest time period in primary dentition during which changes in caries increment usually occur [54]. In spite of this short period, the caries increment was significantly lower in the intervention group than in the control group. Felden et al. [36] and their study from Brazil, had a follow-up period of 3 years. When children were 4 years old, they evaluated the long-term effectiveness of a nutritional program given to mothers during the first year of the child’s life. As both caries incidence and severity of caries was a less problem in the intervention group than in the control group, the program was found to be effective.

## Conclusions

On the basis of this review, we maintain that in addition to studies of water fluoridation and fluoride toothpaste, there are other preventive intervention studies providing scientific evidence for caries reduction among children and adolescents with immigrant or low socioeconomic backgrounds. Supervised toothbrushing for 5-year-olds in schools was found to be an effective prevention technique for use in underprivileged groups, but also studies with a child/mother approach from very early, targeting nutrition and broad oral health education of mothers. For older children, a slow-release fluoride device and application of an APF gel have been shown to be effective.
